# 

**Published:** 1858-01

**Authors:** 


### CONTENTS OF THE JANUARY NUMBER.

### ORIGINAL COMMUNICATIONS.

Page

ART. I.—Fractures of the Inferior Maxilla. By Frank H. Hamilton, M, D., - - - - - 449

ART. II.—Blood Poisoning. By J. T. Jameson, M. D., - - - - - - - - - - - 462

ART. III.—Symptoms, Pathology, and Treatment of Dissecting Wounds. By E. R. Maxson, M.D. 572

ART. IV.—Reports of Cases occurring in the Medical Ward of the Buffalo Hospital. Reported by Dr. A. W. Nichols, - - - - - - - - - - - - - - - - - - 472

ART. V.—Abstract of the Proceedings of the Buffalo Medical Association, - - - - - 478

ART. VI.—The Transactions of the American Medical Association. Instituted 1847. Vol. X, - 492

ART. VII.—Monthly Record of Deaths in the City of Buffalo. By C. L. Dayton, M.D., Health Physician, - - - - - - - - - - - - - - - - - - - - 501

### EDITORIAL DEPARTMENT.

Sydenham Society, - - - - - - - - - - - - - - - - - 503

Sorghum, or Chinese Sugar Cane, - - - - - - - - - - - - - - - 504

The New Year and Medical Journals, - - - - - - - - - - - - - 509

Honors conferred upon American Physicians, - - - - - - - - - - - 509

Buffalo General Hospital, - - - - - - - - - - - - - - - - - 512

